# The Effects of Stand Density on the Growth of Winter Rapeseed in an Agroforestry Intercropping System in the Red Soil Slopes of Southern China

**DOI:** 10.3390/plants14091374

**Published:** 2025-05-01

**Authors:** Xin Luo, Xianghui Lu, Haina Zhang, Haolong Wan, Yue Zhang, Xiaoying Feng

**Affiliations:** 1 Jiangxi Camphor Tree Breeding and Development Engineering Research Center, Nanchang Institute of Technology, Nanchang 330099, China; 19979946654@163.com (X.L.); zhanghaina@nit.edu.cn (H.Z.); w672617961@163.com (H.W.); 15029534140@163.com (Y.Z.); fengxx@gmail.com (X.F.); 2 School of Soil and Water Conservation, Nanchang Institute of Technology, Nanchang 330099, China

**Keywords:** agroforestry system, winter rapeseed, camphor dwarf forest, stand density, slope

## Abstract

Agroforestry systems can improve land use efficiency and increase the output of agricultural and forestry products. In this study, a camphor forest–winter rapeseed composite system was used as the research object from 2023 to 2024. A randomized block experiment was used to set different slopes, S1, S2, and S3 (5°, 10°, and 15°), and camphor forest densities D1, D2, and D3 (row spacing of 1.5 m × 1.5 m, 1.0 m × 1.5 m, 1.0 m × 1.0 m) to compare a single crop (CK) of winter rapeseed and analyze its growth status. This study showed that slope and camphor forest density significantly affected the growth indicators of winter rapeseed. Among the intercropping treatments, S1D2 (5°, 1.0 m × 1.5 m) performed best. In the late growth period of winter rapeseed (flowering to maturity), the treatment increased leaf area index, relative chlorophyll content, root system indicators (length, surface area, volume), theoretical yield, and actual yield, and it increased the aboveground biomass per unit area. Although the actual yield of intercropping on slopes S1, S2, and S3 was 2.52%, 2.82%, and 1.72% lower than that of monocropping, respectively, the ground surface was exposed and idle in winter after the camphor trees were cut down in September. Intercropping winter rapeseed with camphor trees can improve land utilization and increase surface coverage. The results showed that the S1D2 (5°, 1.0 m × 1.5 m), S2D1 (10°, 1.5 m × 1.5 m), and S3D1 (15°, 1.5 m × 1.5 m) treatments performed well in terms of biomass accumulation and yield, and they can be used as recommended intercropping patterns for different slopes.

## 1. Introduction

In recent years, with the acceleration of global population growth and urbanization, land resources have become increasingly limited, and innovative and efficient planting and land use models are urgently needed. Agroforestry integrates agriculture and forestry. It breaks through the limitations of traditional agriculture and forestry, integrating trees and crops to achieve efficient resource utilization and coordinated ecological development. The implementation of this system in different terrains is of great significance to land resource utilization, the economic benefits of agricultural and forestry products, and the prevention and control of soil erosion on slopes [1,2,3].

The ecological benefits of agroforestry systems are the foundation of their cultivation performance and effectiveness. On the one hand, they can increase land surface coverage and reduce soil erosion [4]; on the other hand, they can ease the conflict between agriculture and forestry for land and increase output per unit area of land [5]. Their cultivation performance and effectiveness mainly depend on the growth of plants. Because they can directly reflect the effectiveness of the system, domestic and foreign scholars have conducted much research on this from various angles. (1) On the topic of changing light and shading conditions, for example, Surki et al. [6] found that the growth rate of wheat and barley reached the maximum 2.5 m away from an almond tree, and the growth rate was 35% and 39% higher than that of single crops on their own; Huang Jianxiong et al. [7] studied a rubber forest composite system and showed that there were significant differences in crop growth under light deficiency conditions. (2) On the topic of tree age, Souza et al. [8] found that in four agroforestry systems of different ages, the soil water storage of the three younger systems was higher, and the average pH value was also better. (3) On water conditions, Jie Tingting et al. [9] studied the decomposition characteristics of poplar leaves and corn straw litter under different water stresses and found that as drought intensified, the mass residual rate of both increased, and the decomposition rate decreased. (4) On management measures, Fitra et al. [10] changed different management systems in a coffee pine agroforestry system, such as pruning, adding fertilizers or a combination of organic and inorganic fertilizers, and they concluded that the combination of pruning and fertilization was the best for improving coffee bean yield. (5) On soil surface coverage, Kun et al. [11] covered winter wheat straw before sowing rice between willow rows and found that the organic matter content of the soil in the covered treatment was significantly higher than that in the uncovered treatment, which was beneficial to crop growth. (6) On soil type, Zhao et al. [12] found that millet–soybean intercropping had the best growth effect on mixed soil among three types of soils: loess, sandy soil, and mixed soil. Liu et al.’s [13] study on the intercropping of tomatoes and Sedum sedum Hance showed that the yield of tomatoes planted in four soils was ranked as follows: Zhejiang Fuyang Chao soil (ZJ) > Heilongjiang Harbin black soil (DB) > Hunan Liling red soil (HN) > Shandong Shouguang Chao soil (SD). (7) On intercrop types, Kokila et al. [14] analyzed seven agroforestry systems and pointed out that selecting suitable tree species and regulating canopy density are the keys to optimizing soil moisture. (8) On crop planting density, Huo Gaopeng [15] revealed the inter-species water competition mechanism by intercropping rapeseed with different densities under apple trees; Narteh et al. [16] studied rubber–plantain intercropping and found that the soil C, N, and P contents of the high-density configuration (three rows of plantain + one row of rubber) were the highest, significantly better than monocropping.

In general, agroforestry has significant advantages in resource utilization efficiency, but its effect is affected by multiple factors such as light, water, crop types, planting density, management measures, tree age, and surface cover. However, existing studies have paid insufficient attention to the combined effects of stand density and slope conditions. Stand density can improve the microclimate in the forest by regulating the spatial distribution of light, heat, and water, thereby affecting crop growth, root development, and yield formation [17,18,19]. The distribution of soil nutrients and water varies significantly under different slopes. Although suitable slopes are conducive to crop resource acquisition, China’s arable land resources are limited, and it is necessary to explore suitable agroforestry models in different slope areas to avoid ecological risks [20,21].

Camphor (*Cinnamomum camphora* (L.) Presl) is a typical evergreen tree in the southern red soil region. Its spice raw material forest (aromatic camphor) adopts a dwarf forest operation mode, germinating in April each year and being harvested in early October to leave stumps [22,23]; winter rapeseed (*Brassica napus* L.) accounts for 90% of the country’s winter rapeseed planting area, sowing in late October and being harvested in early May of the following year [24]. The growth cycles of the two are staggered, and both require single fertilization. Intercropping rapeseed after camphor is harvested can make use of winter’s idle fields, and its residual roots and fallen leaves provide a base fertilizer for rapeseed seedlings; the stubble after rapeseed is harvested provides nutrients for camphor germination, and research by Wang Jinping et al. [25] has shown that brassinolide produced during rapeseed growth can promote the photosynthesis efficiency of camphor. Therefore, the intercropping model of a dwarf camphor forest and winter rapeseed can improve land productivity and surface coverage and reduce soil erosion through the mechanism of “nutrient cycle-seasonal complementarity-ecological synergy” [26]. It is worth noting that previous studies have not yet conducted research on the effects of different slopes and camphor tree forest densities on agroforestry systems. This study took winter rapeseed as the object, analyzed the growth differences in winter rapeseed under different slopes (5°, 10°, 15°) and camphor tree forest densities (1.5 m × 1.5 m, 1.0 m × 1.5 m, 1.0 m × 1.0 m), and explored the mechanism of the influence of topography and forest structure on crops, aiming to fill the research gap in this field and provide a scientific basis for optimizing the configuration model of agroforestry systems and achieving synergy between economic and ecological benefits in the southern red soil region.

## 2. Materials and Methods

### 2.1. Overview of the Study Area

This study was conducted in the Ecological Science and Technology Park of Nanchang Institute of Technology, Nanchang, Jiangxi Province (116°03′41.18′′ E, 28°41′30.34′′ N). The region has a subtropical humid monsoon climate, with an average annual rainfall of 1500–1700 mm, an average annual temperature of about 18 °C, a maximum temperature of 42 °C, a minimum temperature of −10 °C, average annual sunshine of 1680–1800 h, and sufficient sunlight. The soil texture of the experimental area is a typical southern red soil, developed from Quaternary red clay, with a pH value of about 5.47 and a bulk density of about 1.31 g/cm^3^. The soil pH was determined by the electrometer method [27,28], and the soil bulk density was determined by the ring knife method [29].

### 2.2. Test Materials

The camphor tree variety was “Ganfang No. 1” (two-year-old, cutting seedlings), and the management method was dwarf dense planting and ultra-short rotation felling. The above-ground branches and leaves were harvested in early October every year for camphor essential oil extraction. After felling, the stumps were left at 25 cm, and new branches sprouted and grew in April of the following year. The winter rapeseed variety was “Sunshine No. 131”, which was sown between the rows of the camphor dwarf forest that had been harvested and stumped in late October, harvested in early May of the following year, and the straw was returned to the field.

### 2.3. Experimental Design

**Figure 1:**
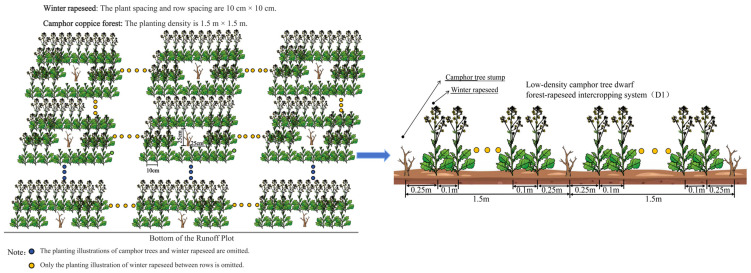
Schematic diagram of test layout (taking S1D1 as an example).

The experiment was set up with three different slopes of 5° (S1), 10° (S2), and 15° (S3). Under each slope, there were three standard runoff plots (20 m long and 5 m wide) with sunny slopes. The test soils in the plots were all typical red soils in the south. The experiment adopted a randomized complete block design (RCBD), and set up winter rapeseed monocropping (CK), low-stand-density camphor dwarf forest–winter rapeseed intercropping system (D1), medium-stand-density camphor dwarf forest–winter rapeseed intercropping system (D2), and high-stand-density camphor dwarf forest–winter rapeseed intercropping system (D3). Winter rapeseed had a plant spacing of 10 cm in both monocropping and intercropping treatments, and rapeseed was planted 25 cm away from the camphor trees in intercropping. There were 12 treatments in total, and each treatment was repeated 3 times in the plot (Figure 1, Table 1). The sowing date of winter rapeseed was 25 October 2023, and the soil was manually turned before sowing to a depth of 20 cm. The three runoff plots under each slope corresponded to three different stand density treatments and winter rapeseed single cropping treatments in turn.

According to the growth period of winter rapeseed, the entire research stage was divided into the seedling stage (6 December to 25 December), wintering stage (25 December to 25 January of the following year), bud stage (25 January of the following year to 25 February), flowering stage (25 February to 25 March), and maturity stage (25 March to 10 May). No base fertilizer was applied before sowing winter rapeseed, and 250 kg/hm^2^ compound fertilizer was applied after rain on 18 December. Rain-fed agriculture, manual weeding, and other field management were consistent with local farmland.

### 2.4. Measurement Items and Methods

During the main growth period of winter rapeseed (seedling stage, wintering stage, bud stage, flowering stage, and maturity stage), the growth and physiological indicators of each treatment, as well as the post-harvest yield, were measured.

(1) Leaf area index, relative chlorophyll content, aboveground biomass, and root parameters: Select nine representative winter rapeseed plants with similar growth from each plot (leaves were not measured during the silique stage due to shedding). Measure crown width with a steel tape to estimate single-plant area. Use a SPAD—502 (Tokyo, Japan) chlorophyll meter to measure the SPAD values of the three top-most leaves on the main stem, taking three readings per leaf and averaging them; then, average the values of the three leaves as the plant’s SPAD value. Conduct destructive sampling; dig out the roots, clean soil, and separate plant organs. Measure individual leaf areas with a Yaxin—1241 (Beijing, China) leaf area meter, calculate the total leaf area, and divide by the single-plant area to obtain the leaf area index. Bag stems and leaves, sterilize at 105 °C for 30 min, dry at 75 °C to constant weight, and measure dry weights of each organ with an electronic balance to calculate aboveground biomass (stems and leaves in early stages; stems, leaves, and fruits at maturity). Wash separated roots, scan with an EPSON Perfection V700 scanner (Beijing, China), and analyze root morphological parameters using WinRhizo Pro Vision 5.0 software.

(2) Winter rapeseed yield and components: At harvest, select three 1 m^2^ areas in each plot and ten plants from each area. Count the number of effective siliques per plant and the number of grains per silique (average of 3 siliques from upper, middle, and lower parts of the main stem). After three-day drying, remove hulls and measure the thousand-grain weight. Calculate theoretical yield as single-plant yield multiplied by density; the actual yield is the dried grain weight of sampled plants from each plot.

### 2.5. Statistical Analysis

This paper uses Excel 2016 for basic arrangement of experimental data; SPSS Statistics 26.0 was used for two-way ANOVA (used to analyze the individual effects of each factor and the interactions between factors), the least significant difference method (LSD) [30] was used for multiple comparisons (used to determine whether there were significant differences between treatments), and the Pearson correlation analysis [31] was used (used to analyze the linear correlation between two quantitative variables). Origin 2021 was used for plotting, including the changes in aboveground biomass of winter rapeseed at different growth stages, the changes in relative chlorophyll content, the changes in leaf area index, the changes in root characteristics, and the heat map of correlation analysis of various indicators at maturity.

## 3. Results

### 3.1. Effects of Intercropping Patterns on the Aboveground Growth of Winter Rapeseed at Different Growth Stages

**Figure 2:**
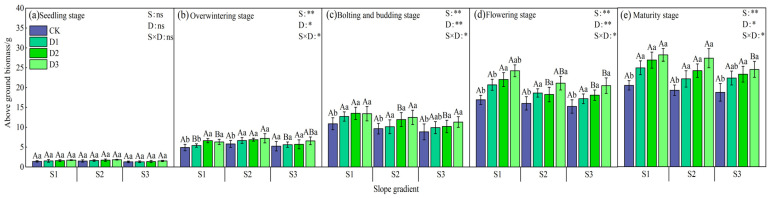
Changes in aboveground biomass of individual rapeseed plants at different growth stages under different intercropping patterns. S1, S2, and S3 represent slopes of 5°, 10°, and 15°, respectively; CK, D1, D2, and D3 represent single cropping of rapeseed in the control experiment (winter rapeseed density 1 million Plant/ha), camphor tree forest density 1.5 m × 1.5 m (winter rapeseed 850,000 Plant/ha), 1.0 m × 1.5 m (winter rapeseed 800,000 Plant/ha), and 1.0 m × 1.0 m (winter rapeseed 700,000 Plant/ha) intercropping, respectively. The lowercase letters in the columns represent the significant levels of the intercropping and control treatments at different stand densities (LSD, *p* < 0.05), and the uppercase letters represent the significant levels of the slope treatments (LSD, *p* < 0.05). ns indicates no significant difference, * indicates a significant difference at the *p* < 0.05 level, and ** indicates a significant difference at the *p* < 0.01 level.

The change pattern of aboveground biomass of winter oilseed plants under different intercropping patterns is mainly as follows (Figure 2): Under the same slope treatment, the biomass of individual plants in the D3 treatment (1.0 m × 1.0 m) in the early and middle growth stages was the highest, and there were significant differences among the D1, D2, and D3 treatments in the bud stage (*p* < 0.05), but no significant differences in the late growth stages; under the same stand density, the biomass of the S2 slope treatment in the early growth stages was higher than that of the S1 and S3 treatments, and in the middle and late stages, S1 > S2 > S3. In the flowering stage, only the S1 slope D2 treatment had significant differences with the S2 and S3 treatments, and the S1 and S2 slope treatments were significantly higher than the S3 treatments in the mature stage (*p* < 0.05). In addition, there was no significant difference in the biomass of individual plants between different slopes under the CK treatment. The data of the maturity period showed that under the same stand density, the biomass of the S1 slope treatment was 18.75% and 33.49% higher than that of S2 and S3 on average; under the same slope, the yield of the D1, D2, and D3 treatments increased by 25.95%, 28.81%, and 34.60%, respectively, compared with the CK monoculture.

**Figure 3:**
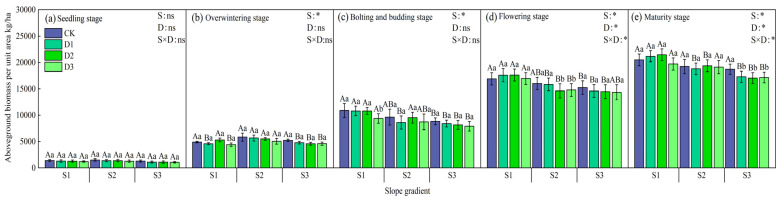
Changes in aboveground biomass per unit area of rapeseed at different growth stages under different intercropping patterns. S1, S2, and S3 represent slopes of 5°, 10°, and 15°, respectively; CK, D1, D2, and D3 represent single cropping of rapeseed in the control experiment (winter rapeseed density 1 million Plant/ha), camphor tree forest density 1.5 m × 1.5 m (winter rapeseed 850,000 Plant/ha), 1.0 m × 1.5 m (winter rapeseed 800,000 Plant/ha), and 1.0 m × 1.0 m (winter rapeseed 700,000 Plant/ha) intercropping, respectively. The lowercase letters in the columns represent the significant levels of the intercropping and control treatments at different stand densities (LSD, *p* < 0.05), and the uppercase letters represent the significant levels of the slope treatments (LSD, *p* < 0.05). ns indicates no significant difference, * indicates a significant difference at the *p* < 0.05 level.

The biomass of winter oilseed per unit area showed significant differences under different intercropping patterns (Figure 3). In the early and middle stages of growth, the biomass of the monoculture treatment (CK) was the highest. Among the camphor tree forest density treatments, D1 > D2 > D3, but there was no significant difference with CK. During the flowering period, CK was still the highest in the S2 and S3 slopes, and the S1 slope D2 treatment performed best. During the maturity period, S1 slope D2 was the best; S2 slope D2 was significantly higher than other treatments; and S3 slope CK was significantly higher than each forest density treatment (*p* < 0.05). Under the same forest density, the S2 slope had the highest biomass in the early stage of growth, and S1 > S2 > S3 in the middle and late stages. Data from the maturity period showed that the biomass of the S1 slope was 8.62% and 12.65% higher than S2 and S3 on average under the same density; S1 slope D2 treatment increased yield by 18.45%, 8.17%, and 10.81% compared with CK, D1, and D3, respectively.

**Figure 4:**
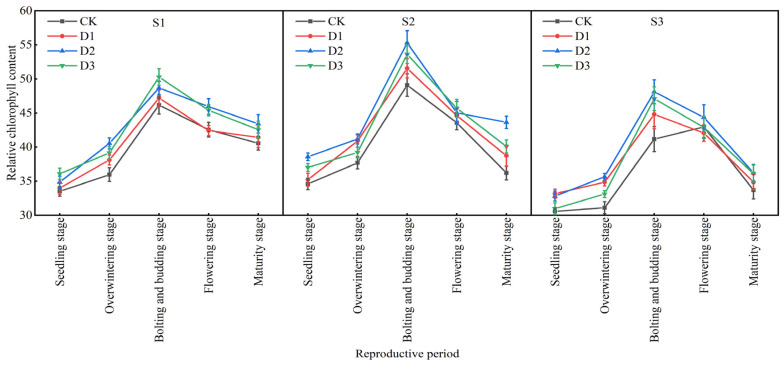
Changes in the relative chlorophyll content of rapeseed during different growth stages under different intercropping patterns. S1, S2, and S3 represent slopes of 5°, 10°, and 15°, respectively; CK, D1, D2, and D3 represent single cropping of rapeseed in the control experiment (winter rapeseed density 1 million Plant/ha), camphor tree forest density 1.5 m × 1.5 m (winter rapeseed 850,000 Plant/ha), 1.0 m × 1.5 m (winter rapeseed 800,000 Plant/ha), and 1.0 m × 1.0 m (winter rapeseed 700,000 Plant/ha) intercropping, respectively.

The changing patterns of relative chlorophyll content of winter rapeseed under different intercropping treatments are mainly shown as follows (Figure 4): The leaf area index and leaf SPAD value of winter rapeseed first increase and then decrease with the growth period, drop sharply during the flowering period, and decrease again during the maturity period; under the same slope, SPAD values of the S2 and S3 slopes first increase and then decrease with the increase in stand density, reaching the maximum value in D2 treatment; the SPAD value of the S1 slope increases with the increase in density in the early and middle growth stages, reaches the peak value in D3 treatment, and the later changes are consistent with S2 and S3; under the same stand density, the SPAD value of S2 slope treatment in the early and middle growth stages is significantly higher than that of S1 and S3 slopes; and SPAD values of treatments during each growth period are higher than those of single cropping, and the differences are most significant in the bud stage and maturity stage. Overall, the SPAD value of the S2 slope is higher, the SPAD value of the S3 slope is lower, the change amplitude of the S1 slope is smaller, and the changes in S2 and S3 slopes are obvious.

**Figure 5:**
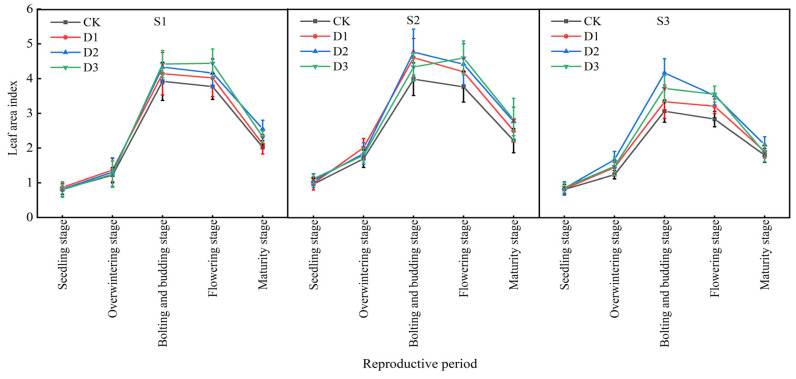
Changes in the leaf area index of rapeseed during different growth stages under different intercropping patterns. S1, S2, and S3 represent slopes of 5°, 10°, and 15°, respectively; CK, D1, D2, and D3 represent single cropping of rapeseed in the control experiment (winter rapeseed density 1 million Plant/ha), camphor tree forest density 1.5 m × 1.5 m (winter rapeseed 850,000 Plant/ha), 1.0 m × 1.5 m (winter rapeseed 800,000 Plant/ha), and 1.0 m × 1.0 m (winter rapeseed 700,000 Plant/ha) intercropping, respectively.

Overall, the leaf area index (LAI) of winter rapeseed in different intercropping treatments showed a trend of increasing first and then decreasing with the extension of the growth period. The S3 slope treatment reached its maximum value at the bud stage, and the D3 and CK treatments of the S1 and S2 slopes reached their peak values at the flowering stage. The LAI dropped sharply at the maturity stage due to a large amount of leaf fall (Figure 5). Under the same intercropping stand density, the LAI of winter rapeseed first increased and then decreased with the increase in slope. Among them, the LAI of the S2 slope treatment increased significantly in each growth period, such as an increase of 12.96% to 20.45% in the seedling stage. Under the same slope, the LAI of intercropping treatments was better than that of monocropping. The LAI of winter rapeseed on the S1 and S2 slopes increased with the increase in camphor intercropping density. For example, the LAI of D3 treatment at maturity on the S1 slope was 1.13% higher than that of single-crop CK; the LAI of winter rapeseed at each growth stage on the S3 slope increased first and then decreased with the increase in camphor intercropping density, among which the LAI of D2 treatment at maturity was 15.38% higher than that of CK.

### 3.2. Effects of Intercropping on Root Characteristics of Winter Rapeseed at Different Growth Stages

**Figure 6:**
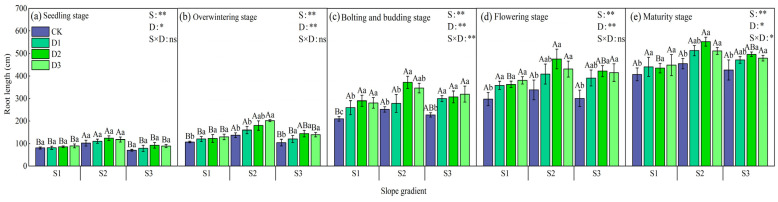
Changes in the root length of rapeseed during different growth stages under different intercropping patterns. S1, S2, and S3 represent slopes of 5°, 10°, and 15°, respectively; CK, D1, D2, and D3 represent single cropping of rapeseed in the control experiment (winter rapeseed density 1 million Plant/ha), camphor tree forest density 1.5 m × 1.5 m (winter rapeseed 850,000 Plant/ha), 1.0 m × 1.5 m (winter rapeseed 800,000 Plant/ha), and 1.0 m × 1.0 m (winter rapeseed 700,000 Plant/ha) intercropping, respectively. The lowercase letters in the columns represent the significant levels of the intercropping and control treatments at different stand densities (LSD, *p* < 0.05), and the uppercase letters represent the significant levels of the slope treatments (LSD, *p* < 0.05). ns indicates no significant difference, * indicates a significant difference at the *p* < 0.05 level, and ** indicates a significant difference at the *p* < 0.01 level.

Different combinations of slopes and stand densities had significant effects on the root length of winter rapeseed (Figure 6). The root length of winter rapeseed showed differences with the growth period. Except for the seedling stage, the root length of each growth period treatment was significantly higher than that of monoculture (*p* < 0.05). In the early growth stage, with the increase in camphor intercropping density, the root length of the S1 and S2 slopes showed a trend of D3 > D2 > D1 > CK, the S3 slope was D2 > D3 > D1 > CK, and the root length of the S2 slope was significantly higher than that of S1 and S3 slopes; in the bud stage, the root length of the S1 and S2 slopes showed D2 > D3 > D1 > CK, the S3 slope was D3 > D2 > D1 > CK, and the root length of S1 and S2 slopes treated with D2 and D3 intercropping was significantly higher than that of monoculture (*p* < 0.05); and in the late growth stage, the change patterns of each slope tended to be consistent, the S1 slope was D3 > D2 > D1 > CK, and the S2 and S3 slopes were D2 > D3 > D1 > CK. Under the same stand density, the root length of winter rapeseed in the S2 slope at each growth stage was significantly higher than that in the S1 and S3 slopes. The data at maturity showed that the root length of S1 slope D3 treatment increased by 11.23%, 4.47%, and 8.55% compared with CK, D2, and D1, respectively; the root length of S2 and S3 slope D2 treatment increased by an average of 10.11% compared with CK.

**Figure 7:**
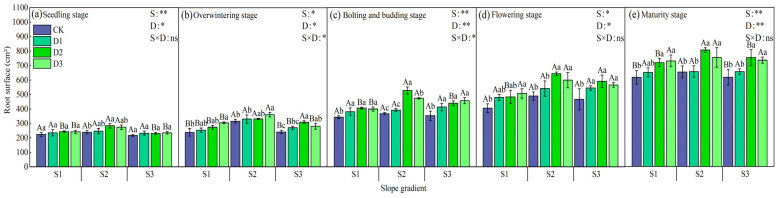
Changes in the root surface area of rapeseed during each growth period under different intercropping patterns. S1, S2, and S3 represent slopes of 5°, 10°, and 15°, respectively; CK, D1, D2, and D3 represent single cropping of rapeseed in the control experiment (winter rapeseed density 1 million Plant/ha), camphor tree forest density 1.5 m × 1.5 m (winter rapeseed 850,000 Plant/ha), 1.0 m × 1.5 m (winter rapeseed 800,000 Plant/ha), and 1.0 m × 1.0 m (winter rapeseed 700,000 Plant/ha) intercropping, respectively. The lowercase letters in the columns represent the significant levels of the intercropping and control treatments at different stand densities (LSD, *p* < 0.05), and the uppercase letters represent the significant levels of the slope treatments (LSD, *p* < 0.05). ns indicates no significant difference, * indicates a significant difference at the *p* < 0.05 level, and ** indicates a significant difference at the *p* < 0.01 level.

There were significant differences in the root surface area of winter rapeseed under each treatment (*p* < 0.05). During different growth periods, the root surface area of intercropping treatments on each slope was better than that of single cropping treatments (Figure 7). In the seedling stage, the root surface area of winter rapeseed was D3 > D2 > D1 > CK with the change in camphor tree stand density. In the overwintering period, the overall performance was consistent with the seedling stage, and the root surface area of each treatment increased compared with the seedling stage. In the bud stage, the performance of the S2 and S3 slopes was the same as the previous two stages, and the S1 slope changed to D2 > D3 > D1 > CK. There were significant differences among the treatments on S2. The intercropping of camphor tree D2 stand density and D3 stand density increased significantly and increased extremely significantly compared with other treatments (*p* < 0.05). In the flowering period, the root surface area of each slope increased significantly, all of which were D3 > D2 > D1 > CK, and D3 increased by an average of 10.58% compared with other treatments. At maturity, D3 > D2 > D1 > CK on S1, and D3 increased by 14.11% on average compared with other treatments; the slope change pattern of S2 and S3 was D2 > D3 > D1 > CK, and D2 increased by 11.18% (S2) and 19.58% (S3) on average compared with other treatments. Under the same stand density, the root surface area of winter rapeseed at each growth stage was the largest on slope S2.

**Figure 8:**
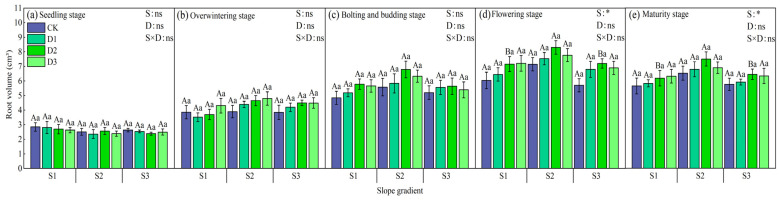
Changes in the root volume of rapeseed during each growth period under different intercropping patterns. S1, S2, and S3 represent slopes of 5°, 10°, and 15°, respectively; CK, D1, D2, and D3 represent single cropping of rapeseed in the control experiment (winter rapeseed density 1 million Plant/ha), camphor tree forest density 1.5 m × 1.5 m (winter rapeseed 850,000 Plant/ha), 1.0 m × 1.5 m (winter rapeseed 800,000 Plant/ha), and 1.0 m × 1.0 m (winter rapeseed 700,000 Plant/ha) intercropping, respectively. The lowercase letters in the columns represent the significant levels of the intercropping and control treatments at different stand densities (LSD, *p* < 0.05), and the uppercase letters represent the significant levels of the slope treatments (LSD, *p* < 0.05). ns indicates no significant difference, * indicates a significant difference at the *p* < 0.05 level.

The root volume of winter rapeseed under different treatments was significantly different (*p* < 0.05). At the same slope, in the seedling stage, the root volume on the S1 slope was CK > D1 > D2 > D3, and CK increased by 6.15% on average compared with other treatments; on the S2 slope, it was D2 > CK > D1 > D3, and D2 increased by 5.87% on average; and on the S3 slope, it was D3 > CK > D1 > D2, and D3 increased by 5.12% on average. At this time, there was no obvious pattern in the root volume (Figure 8). During the wintering period, the slopes of S1 and S2 were D3 > D2 > D1 > CK, and the slope of S3 was D3 > CK > D2 > D1. The intercropping of camphor trees with high stand density (D3) performed best. At the bud stage, it was D3 > D2 > D1 > CK on all slopes. During the flowering period, it was D2 > D3 > D1 > CK overall, and the root volume of different intercropping patterns was significantly different (*p* < 0.05). At maturity, the root volume changes at each slope were consistent with those at flowering, and the intercropping of camphor trees with medium stand density (D2) performed best, increasing by 9.08% (S1), 15.41% (S2), and 10.58% (S3) on average compared with other treatments. Under the same stand density, the root volume of winter rapeseed at each growth stage was the largest on slope S2.

**Figure 9:**
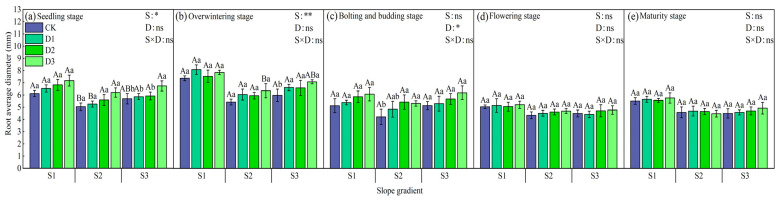
Changes in the root average diameter of rapeseed at different growth stages under different intercropping patterns. S1, S2, and S3 represent slopes of 5°, 10°, and 15°, respectively; CK, D1, D2, and D3 represent single cropping of rapeseed in the control experiment (winter rapeseed density 1 million Plant/ha), camphor tree forest density 1.5 m × 1.5 m (winter rapeseed 850,000 Plant/ha), 1.0 m × 1.5 m (winter rapeseed 800,000 Plant/ha), and 1.0 m × 1.0 m (winter rapeseed 700,000 Plant/ha) intercropping, respectively. The lowercase letters in the columns represent the significant levels of the intercropping and control treatments at different stand densities (LSD, *p* < 0.05), and the uppercase letters represent the significant levels of the slope treatments (LSD, *p* < 0.05). ns indicates no significant difference, * indicates a significant difference at the *p* < 0.05 level, and ** indicates a significant difference at the *p* < 0.01 level.

There were differences in the average root diameter of winter rapeseed under each treatment. In the seedling stage, the overall trend was D3 > D2 > D1 > CK, and D3 increased by 6.33% on average compared with other treatments (Figure 9). In the wintering period, the average root diameter increased slightly, with D1 > D3 > D2 > CK on the S1 slope and D3 > D1 > D2 > CK on the S2 and S3 slopes. In the bud stage, the slope pattern was D3 > D2 > D1 > CK, and D3 increased by 6.12% on average compared with other treatments, which was similar to the seedling stage. As the growth period progressed, the average root diameter decreased overall, and the roots tended to grow more vertically. In the flowering period, D3 > D2 > D1 > CK was found on different slopes, and D3 intercropping had obvious advantages in high forest density. Compared with the flowering period, the average root diameter did not change significantly in the maturity period, and the change pattern of each treatment was similar, with D3 intercropping still being the best. In the late growth period (flowering period and maturity period), the changes in each slope treatment were not large. Overall, the average root diameter was better on slope S1 and lowest on S2, which was opposite to the changes in root length, root surface area, and root volume at different slopes.

### 3.3. Effects of Intercropping Patterns on Winter Rapeseed Yield Components and Yield

The yield components of winter rapeseed were different under different intercropping conditions (Table 2). At the same slope, the number of siliques and thousand-grain weight on S1, S2, and S3 were all D3 > D2 > D1 > CK. D3 increased by 27.4% (S1), 26.2% (S2), and 31.4% (S3) compared with CK; the thousand-grain weight under D3 treatment increased by 2.72% (S1), 2.73% (S2), and 2.77% (S3) compared with CK. In terms of the number of single siliques, D2 > D3 > D1 > CK on S1, and D2 increased by 5.50%, 3.24%, and 0.56% compared with D1, D3, and CK, respectively; D3 > D2 > D1 > CK on S2 and S3, and D3 increased by 7.78% (S2) and 6.35% (S3) compared with CK. In general, the yield components on each slope increased with the increase in camphor tree forest density. The three intercropping treatments were better than monocropping, and the number of effective siliques per plant under intercropping treatments on different slopes was significantly higher than that under monocropping (*p* < 0.05).

In terms of yield, different intercropping treatments had a significant effect on winter rapeseed yield (*p* < 0.05). In terms of theoretical yield, monocropping (CK) had the highest yield per unit area under the same slope; the intercropping density of D2 on slopes S1 and S3 increased yield by 4.09% and 5.00% on average compared with D1 and D3, and the average yield of D1 on slope S2 increased by 1.81% on average compared with D2 and D3; under different slopes, monocropping increased yield by 3.58% (S1), 5.17% (S2), and 5.35% (S3) on average compared with intercropping, and there was no significant difference in intercropping treatments on different slopes; and under the same treatment, the theoretical yield of slope S1 was the highest, 8.68% and 13.43% higher than that of slopes S2 and S3, respectively, and significantly higher than that of slope S3 (*p* < 0.05). The actual yield was significantly lower than the theoretical yield, and the change pattern was different on each slope. The average yield of D1 on the S1 slope was 5.34% higher than that of D2 and D3, and the average yield of D1 on the S2 and S3 slopes was 2.89% and 6.28% higher than that of D2 and D3. On different slopes, the average yield of monocropping was 12.71% (S1), 7.62% (S2), and 8.38% (S3) higher than that of intercropping. The actual yield of each treatment on the S1 and S3 slopes was significantly different (*p* < 0.05). Under the same treatment, the actual yield of the S1 slope was the highest, which was 5.02% and 12.28% higher than that of S2 and S3, respectively, and significantly higher than that of the S2 and S3 slopes (*p* < 0.05).

### 3.4. Correlation Analysis of Various Indicators During Winter Rapeseed Maturity

**Figure 10:**
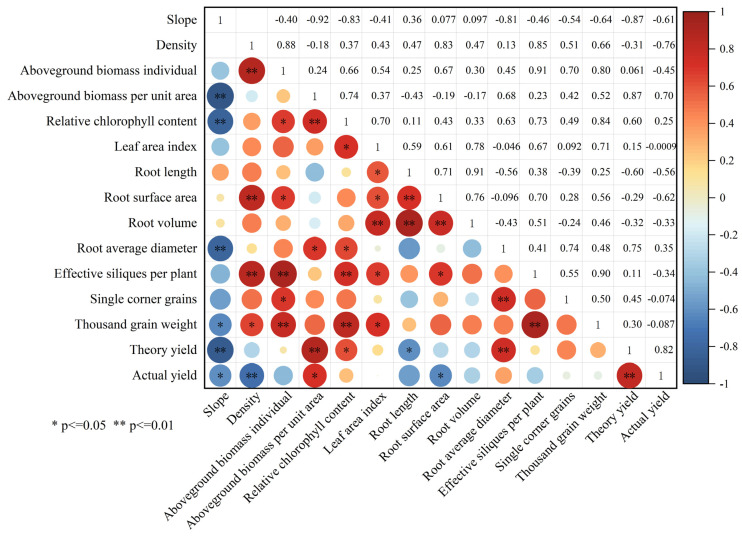
Correlation heat map of various indicators of winter rapeseed at maturity. Correlation coefficient >0 indicates positive correlation, and <0 indicates negative correlation. * indicates a significant difference at the *p* < 0.05 level, and ** indicates a significant difference at the *p* < 0.01 level.

The results showed that there were correlations among multiple indicators of winter rapeseed (Figure 10). Among them, slope was positively correlated only with root length, root surface area, and root volume (*p* < 0.05), negatively correlated with other indicators, and extremely significantly negatively correlated with biomass per unit area, SPAD, average root diameter, and theoretical yield (*p* < 0.01). Camphor tree forest density was negatively correlated with biomass per unit area, theoretical yield, and actual yield and extremely significantly negatively correlated with actual yield (*p* < 0.01); it was positively correlated with other indicators and extremely significantly positively correlated with aboveground biomass per plant, average root diameter, and number of effective siliques per plant (*p* < 0.01). In addition, the number of siliques and 1000-grain weight were all positively correlated with theoretical yield, but the correlation coefficients were low, 0.11, 0.45, and 0.30, respectively, and negatively correlated with actual yield (*p* > 0.05), which may be related to the increase in camphor tree forest density leading to a decrease in rapeseed planting density.

## 4. Discussion

### 4.1. Effect of Intercropping Density on Winter Rapeseed Growth, Root Characteristics, and Yield

The agroforestry model can effectively improve the utilization rate of land and resources per unit area and create higher productivity than pure forest management. However, if the stand density is not properly selected, it will directly affect the productivity and function of the stand itself and the crops in the forest [32,33,34]. In this study, compared with monoculture, winter rapeseed was intercropped at three different stand densities, and the aboveground biomass of each plant increased to varying degrees from the seedling stage to the mature stage, but the aboveground biomass per unit area changed in the opposite way. Only in the flowering and mature stages was the winter rapeseed intercropped with stand densities D1 and D2 on the S1 slope better than the monoculture CK, and the monoculture rapeseed was better on all slopes during the rest of the growth period. This is because intercropping changes the planting density of winter rapeseed. The planting density of winter rapeseed between the rows of camphor tree D1 stand density is 850,000 plants/ha, D2 is 800,000 plants/ha, and D3 is 700,000 plants/ha. As the planting density decreases, the aboveground biomass of each rapeseed plant increases due to the increase in growth space. However, due to the decrease in planting density, the overall number of plants decreased, and the aboveground biomass per unit area on each slope was lower than that of monoculture. During the flowering and maturity periods, the aboveground biomass per unit area of winter rapeseed intercropped with D1 and D2 stand densities on the S1 slope was better than that of monoculture CK. This may be because, on the one hand, the biomass of a single plant continued to increase due to the expansion of growth space, and on the other hand, the planting density of D1 and D2 maintained a certain population size, and the stand environment improved the light, temperature, and humidity conditions, promoted the photosynthesis and material accumulation of winter rapeseed [35], and compensated for the biomass loss caused by the decrease in planting density, thus achieving a reversal. Monoculture still has an advantage over other slopes, which may be due to the interaction between environmental factors such as light and water on different slopes and the stand, and it failed to reach the optimal combination to promote the growth of intercropping biomass. Different stand densities have an impact on multiple growth indicators of winter rapeseed and show stage characteristics in the growth process. At each growth period and slope, the aboveground biomass of a single plant of winter rapeseed intercropped with high-density stands (D3) increased significantly. In terms of aboveground biomass per unit area, the best results were obtained when the oil seedlings were planted in the seedling stage, wintering stage, and bud stage, and the results were significantly improved when the oil seedlings were planted in the medium-density stand (D2) during the flowering and maturity stages. It can be seen that plant growth is a complex dynamic process, and environmental factors are interrelated and influence each other.

Plant leaves are responsible for transpiration and photosynthesis and are crucial to plant growth and development. Leaf area index is a key indicator for measuring the growth status of plant populations [36], and chlorophyll is a core element that reflects plant photosynthetic efficiency and physiological status [37]. In this study, the leaf area index of winter rapeseed increased most significantly during the bud stage, and the relative chlorophyll content increased the most during the bud and flowering stages. The study also showed that in most treatments and during each growth period, the relative chlorophyll content of winter rapeseed had the same trend as the leaf area index, reaching a peak during the bud stage and when intercropped with camphor trees at higher stand densities of D2 and D3, which is consistent with the research conclusions of Wang Han et al. [38] In the intercropping system of forests and farmers, changes in crop yields can directly reflect the effect of intercropping [39]. This study showed that compared with monocropping, the actual yield of winter rapeseed on all slopes decreased under different intercropping conditions, with an average decrease of 8.18%. This is because intercropping reduced the planting density, and the number of plants harvested in each treatment was different, resulting in the overall actual yield being lower than that of monocropping. Moreover, the intercropping conditions at different stand densities were different. For example, on slope S1, the actual yield was ranked D2 > D1 > D3, while on S2 and S3, it was D1 > D2 > D3. However, when intercropping with high camphor stand density, the number of siliques, number of grains per silique, and 1000-grain weight of rapeseed were significantly higher than those of other treatments, which was similar to the increase in biomass per plant under low density due to improved growth space.

In summary, the density of camphor trees has a significant impact on the growth indicators of winter rapeseed, such as leaf area index, relative chlorophyll content, aboveground biomass per plant and per unit area, actual yield, and its components. The main reason may be that the planting density of winter rapeseed intercropped under different camphor tree stand densities is different, resulting in differences in growth space. On slopes S1 and S2, when winter rapeseed was intercropped with camphor tree stand density D2, the aboveground biomass per unit area was significantly higher, even exceeding the single-crop CK treatment. This is similar to the study of Han Bingbing et al. [40] in the Yang-agriculture system; that is, the medium stand density is most beneficial to the growth of understory crops. On the S1 slope, the actual yield of the D2 treatment was lower than that of the single-crop CK, but higher than that of the D1 and D3 treatments. This change is different from other slopes and may be attributed to the interactive effect of slope and density. However, previous studies have shown that in the agroforestry model, crops are easily affected by the canopy width of trees. The closer the distance to the trees, the stronger the shading effect, which leads to a decrease in productivity and a negative impact [41,42]. For example, Duan Zhiping et al. [43] pointed out that when jujube and wheat are intercropped, the farther away from the jujube trees, the greater the dry matter accumulation and leaf area index of wheat plants, and the various growth indicators of wheat monoculture are better than intercropping. Wang Lin et al.’s [44] research also showed that in the rainy season, soybeans in the agroforestry system are significantly affected by shading, and the farther away from the trees, the better they grow. These conclusions are opposite to the growth of crops in this experiment, which may be related to the dwarf forest operation of camphor trees [45,46]. On the one hand, after the camphor dwarf forest is cut down, the branches and leaves almost stop growing, and only the underground part grows slowly. When winter rapeseed is sown between the rows without canopy cover, it is conducive to the efficient use of light energy by winter rapeseed. On the other hand, the red soil has low fertility and is acidic [47]. The field management during the growth of the camphor dwarf forest ensures sufficient nutrients, which can provide good base fertilizer for the next round of forest or crop growth and create a high-quality growth environment. Finally, the fallen leaves of the camphor trees return to the soil, increasing the soil organic matter, improving the fertility of the red soil to a certain extent, and affecting the transformation and supply of nutrients in the soil.

In the agroforestry system, since the above-ground part of the camphor dwarf forest is cut down, the interaction between its underground part and the root system of winter rapeseed becomes a key factor affecting the growth of winter rapeseed. This experiment shows that the root system indicators of winter rapeseed in different growth stages show different changing patterns under different slopes. In general, the root length, root surface area, and root volume perform best when intercropped at the density of D3 on slope S1, while on the slopes of S2 and S3, the density of D2 performs best. This difference may be due to the environmental characteristics of different slopes. On slope S1, before the camphor trees are cut down, the higher stand density can effectively shade and reduce water evaporation in the forest, creating good soil moisture conditions for the growth of winter rapeseed roots. On the slopes of S2 and S3, water and nutrients are easily lost. The planting density of winter rapeseed at the density of D2 increases to 800,000 plants/hectare, and the group advantage can make up for the resource loss caused by the slope. The interweaving of the roots of more plants not only enhances soil fixation and reduces soil erosion but also improves the comprehensive utilization rate of limited resources. The relatively sparse layout of camphor trees provides a certain space for rapeseed to grow, enabling it to better cope with adverse environments and promote the development of root length, surface area, and volume. In addition, there are obvious differences in water utilization between forest trees and winter rapeseed. Previous studies have shown that forest trees absorb more deep soil moisture [48,49], while winter rapeseed, as a shallow-rooted crop, mainly uses surface soil moisture [15,50,51], and there is less competition between the two. At the same time, the plant growth stage and phenological period also affect their interaction. In the early growth period of winter rapeseed, camphor trees are dormant, the underground part grows slowly, and the soil between rows is loose, which is conducive to the absorption of nutrients by winter rapeseed. However, in the middle and late growth period, the temperature rises, and the underground part of camphor trees begins to become active, forming a competitive relationship with winter rapeseed. Different stand densities will also change the underground space allocation and soil ecosystem functions [52]. Under medium forest density, underground space is abundant, soil ecology is active, and nutrient circulation is efficient; under low forest density, although underground space is large, nutrient circulation efficiency is low [53,54,55]. This experiment also found that the root length, root surface area, and root volume of winter rapeseed intercropping treatment increased with the growth period, but the average root diameter showed the opposite trend. This may be because the underground part of camphor tree occupied a certain space before sowing, causing the winter rapeseed root system to “avoid” it and grow more vertically; it may also be because the 10 cm plant spacing of winter rapeseed limited the extension of the root system in the horizontal direction of the soil layer. This phenomenon is similar to the research results of Zhou Hua et al. [56] on the betel nut–elephant grass composite system.

### 4.2. Effect of Slope on Winter Rapeseed Growth, Root Characteristics, and Yield

The hilly red soil area in the south has complex terrain and extensive sloping land. Therefore, a reasonable agroforestry planting pattern is of great significance for preventing and controlling soil erosion on slopes and improving ecological benefits [57,58,59]. This study shows that under different stand densities, in the early growth period (seedling stage and wintering period), the aboveground biomass of each treatment on S2 increased significantly compared with S1 and S3. However, in the middle and late growth period, the aboveground biomass of each treatment on slope S1 was significantly superior. This is also different from the change in SPAD values of winter rapeseed with slope in each growth period. In the seedling stage and wintering period, the SPAD values of winter rapeseed on slopes S1 and S2 were similar, and the value on S3 was significantly lower. Entering the bud stage, the leaf area index of slope S2 increased significantly compared with S1 and S3. During the flowering and maturity stages, the SPAD values of the treatments on slopes S2 and S3 dropped significantly compared with the bud stage. Although the values of the treatments on the S1 slope also dropped, the drop was smaller than that of S2 and S3. In addition, the leaf area index of winter rapeseed at different growth stages was not much different overall on different slopes. Only during the wintering and bud stages was the leaf area of winter rapeseed on slope S2 the largest, while that on S3 was relatively small. This may be because the angle between the slope and the light of the 10° slope is conducive to rapeseed photosynthesis, increasing the leaf area index. Zhang Yuanyuan et al. [60] pointed out that slope changes significantly affect the nutrient characteristics of the soil where Bermuda grass is grown. The content of each nutrient decreases with increasing slope, and the soil nutrients within 10° are significantly higher than those at 10° and above. This is consistent with the crop growth situation in this paper. In the early growth stage, the aboveground growth indicators of plants on slope S2 were optimal, but from the bud stage to the maturity stage, the growth indicators on the slope S1 were better. This may be due to the increase in rainfall and the high slope that easily caused nutrient loss [61].

Plant roots play an important role in soil fixation. This study showed that under different stand densities, the root length, root surface area and root volume of winter rapeseed at each growth stage increased as the growth stage progressed. Its change trend with slope is opposite to the change in aboveground biomass of winter rapeseed in the later growth period. It performed best at the S2 slope and had a significant effect on slope. This is consistent with the research results of scholars Zhidan et al. [62] that soybean root length, root surface area, and root volume reached their maximum values at a slope of 10°. However, in this experiment, the average root diameter decreased as the growth stage progressed and decreased with increasing slope. There was little difference between the S2 and S3 slopes, and only the S1 slope increased significantly. Moreover, the slope only had a significant effect on the seedling and wintering stages of winter rapeseed, which is contrary to the research of scholars Nong Gan et al. [63], indicating that the average root diameter of wheat reached its maximum value when the slope was 10°. This may be because on the S1 slope, the camphor tree roots disturbed the soil less, and the soil compactness and porosity were conducive to the lateral growth of the winter rapeseed roots, which led to a significant increase in the average root diameter. It may also be because the red soil is heavy and has poor air permeability [64,65], which resulted in the winter rapeseed roots growing better only in the shallow soil layer [66]. On the S2 and S3 slopes, the camphor tree roots had a stronger effect on soil transformation, and gravity caused soil loss and redistribution to be complex. In order to better anchor the plant and absorb limited water and nutrients, the winter rapeseed roots tended to grow in depth, resulting in a decrease in the average root diameter.

In addition to root characteristics, the adaptability of different slopes and intercropping density of camphor trees is also critical to the growth of winter rapeseed. This study shows that when intercropped on different slopes, the growth indicators of winter rapeseed are better than those of single cropping, but the yield is lower than that of single cropping due to the reduced planting density. On slope S1, winter rapeseed showed good performance in various indicators under the condition of medium forest density D2. Compared with other treatments, D2 increased the yield by an average of 6.89% on S1. On slope S2, the yield was the highest when intercropped camphor tree density D1 was used, but the yield difference with D2 was small, and the root indicators were optimal under D2, which may have a better effect in preventing soil erosion. Both can be selected. On slope S3, intercropped camphor tree density D1 was better. Although the number of winter rapeseed plants and aboveground biomass per unit area were the best when intercropped with D2 on S3, and the other indicators were better under the intercropping condition of D3, the actual yield of D1 increased by an average of 4.02% compared with other treatments. This may be because the 15° slope is relatively steep, and rainwater erosion, rapid fertility loss [67,68], and the red soil structure is easily destroyed, further deteriorating the air permeability, resulting in the stunted development of the winter rapeseed root system [69,70]; the growth condition is not as good as in areas with gentler slopes. Under the D1 camphor tree forest density condition, the planting density of winter rapeseed between rows increases, interacting with the camphor tree root system, enhancing the stability of soil aggregates through the rhizosphere effect, which can effectively reduce soil erosion and reduce the negative impact of soil nutrient loss on the growth environment of winter rapeseed.

## 5. Conclusions

(1) Under different slopes, the aboveground biomass of a single plant in intercropping winter oil was better than that of monocropping at all growth stages and increased with the increase in camphor tree forest density (*p* < 0.05). In the early and middle growth stages, the aboveground biomass per unit area of the CK treatment was better; in the late stage, on the S1 and S2 slopes, the D2 treatment performed better, and on the S3 slope, the CK treatment performed better. The biomass per plant and per unit area decreased with increasing slope.

(2) The leaf area index and the relative content of chlorophyll generally decreased after reaching a peak at the bud stage. The D2 or D3 intercropping treatment was better on all slopes. For the slope, the leaf area index was better on the S2 slope, and the relative content of chlorophyll was better on the S1 slope (*p* > 0.05).

(3) The root index of intercropping is better than that of monocropping, and the intercropping D3 treatment is better on all slopes; the root length, root surface area, and root volume are all S2 > S3 > S1, and the average root diameter has the opposite trend. The average root diameter value on the S1 slope is larger and decreases with the growth period.

(4) On the S3 slope, the red soil structure is easily destroyed by rain erosion, and the growth indexes of winter rapeseed are poor. According to the forest stand density and slope adaptation, the high-yield method is selected. The S1D2, S2D1, and S3D1 treatments perform well and can be used as the recommended intercropping mode.

## Figures and Tables

**Table 1:** Experimental treatments.

Deal with	Slope	Intercropping	Winter Rapeseed Plant Spacing × Row Spacing (m)	Camphor Coppice Plant Spacing × Row Spacing (m)	Winter Rapeseed Planting Density Plants/ha
S1D1	5°	Low-density dwarf camphor tree forest–winter rapeseed intercropping system	0.1 m × 0.1 m	1.5 m × 1.5 m	850,000 plants
S1D2	5°	Intercropping system of dwarf camphor tree forest and winter rapeseed in medium stand density	0.1 m × 0.1 m	1.0 m × 1.5 m	800,000 plants
S1D3	5°	High-stand-density dwarf camphor tree forest–winter rapeseed intercropping system	0.1 m × 0.1 m	1.0 m × 1.0 m	700,000 plants
S1CK	5°	Winter rapeseed monoculture	0.1 m × 0.1 m	/	1 million plants
S2D1	10°	Low-density dwarf camphor tree forest–winter rapeseed intercropping system	0.1 m × 0.1 m	1.5 m × 1.5 m	850,000 plants
S2D2	10°	Intercropping system of dwarf camphor tree forest and winter rapeseed in medium stand density	0.1 m × 0.1 m	1.0 m × 1.5 m	800,000 plants
S2D3	10°	High-stand-density dwarf camphor tree forest–winter rapeseed intercropping system	0.1 m × 0.1 m	1.0 m × 1.0 m	700,000 plants
S2CK	10°	Winter rapeseed monoculture	0.1 m × 0.1 m	/	1 million plants
S3D1	15°	Low-density dwarf camphor tree forest–winter rapeseed intercropping system	0.1 m × 0.1 m	1.5 m × 1.5 m	850,000 plants
S3D2	15°	Intercropping system of dwarf camphor tree forest and winter rapeseed in medium stand density	0.1 m × 0.1 m	1.0 m × 1.5 m	800,000 plants
S3D3	15°	High-stand-density dwarf camphor tree forest–winter rapeseed intercropping system	0.1 m × 0.1 m	1.0 m × 1.0 m	700,000 plants
S3CK	15°	Winter rapeseed monoculture	0.1 m × 0.1 m	/	1 million plants

S1, S2, and S3 represent slopes of 5°, 10°, and 15°, respectively; CK, D1, D2, and D3 represent single cropping of rapeseed in the control experiment (winter rapeseed density 1 million Plant/ha), camphor tree forest density 1.5 m × 1.5 m (winter rapeseed 850,000 Plant/ha), 1.0 m × 1.5 m (winter rapeseed 800,000 Plant/ha), and 1.0 m × 1.0 m (winter rapeseed 700,000 Plant/ha) intercropping, respectively.

**Table 2:** Effects of different intercropping treatments on winter rapeseed yield and yield components.

Experimental Treatment		Number of Effective Siliques per Plant/Piece	Number of Single Corner Grains/Piece	Thousand-Grain Weight/g	Theoretical Yield/(kg·hm^−2^)	Actual Yield/(kg·hm^−2^)
S1	CK	84.25 ± 4.16 Ab	16.91 ± 0.66 Aa	3.68 ± 0.24 Aa	5242.7 ± 285.33 Aa	2984.98 ± 135.48 Aa
	D1	90.33 ± 5.09 Aa	17.28 ± 0.81 Aa	3.73 ± 0.12 Aa	4948.8 ± 250.12 Aa	2704.52 ± 115.62 Ab
	D2	97.38 ± 12.06 Aa	17.84 ± 1.43 Aa	3.74 ± 0.13 Aa	5197.7 ± 304.88 Aa	2751.31 ± 99.48 Aab
	D3	107.33 ± 5.85 Aa	17.74 ± 1.06 Aa	3.78 ± 0.22 Aa	5038.1 ± 201.11 Aa	2568.98 ± 138.21 Ab
S2	CK	80.16 ± 5.54 Ab	16.63 ± 0.28 Aa	3.66 ± 0.31 Aa	4879.0 ± 278.92 Aa	2858.46 ± 147.12 ABa
	D1	94.16 ± 3.78 Aa	15.81 ± 2.03 Aa	3.71 ± 0.18 Aa	4694.5 ± 266.41 Aa	2686.54 ± 126.88 Aa
	D2	96.33 ± 7.15 Aa	16.22 ± 1.67 Aa	3.75 ± 0.23 Aa	4687.4 ± 300.77 ABa	2586.49 ± 103.44 Ba
	D3	101.12 ± 10.83 Aa	17.04 ± 1.14 Aa	3.76 ± 0.21 Aa	4535.1 ± 213.17 Aa	2404.71 ± 98.12 Aa
S3	CK	72.33 ± 5.9 Ab	16.06 ± 1.93 Ab	3.64 ± 0.32 Aa	4680.6 ± 318.46 Ba	2687.52 ± 88.37 Ba
	D1	80.12 ± 5.71 Aa	16.36 ± 1.22 Aa	3.61 ± 0.15 Aa	4423.6 ± 222.65 Ba	2481.4 ± 128.18 Aa
	D2	88.33 ± 7.04 Aa	17.02 ± 1.11 Aa	3.65 ± 0.66 Aa	4588.6 ± 289.71 Ba	2389.6 ± 110.64 Ba
	D3	95.06 ± 9.53 Aa	17.08 ± 1.12 Aa	3.71 ± 0.23 Aa	4316.5 ± 190.84 Ba	2255.07 ± 102.11 Bb
Significant analysis						
Slope		ns	ns	ns	*	*
Density		*	*	*	ns	*
S × D		ns	ns	ns	ns	ns

S1, S2, and S3 represent slopes of 5°, 10°, and 15°, respectively; CK, D1, D2, and D3 represent single cropping of rapeseed in the control experiment (winter rapeseed density 1 million Plant/ha), camphor tree forest density 1.5 m × 1.5 m (winter rapeseed 850,000 Plant/ha), 1.0 m × 1.5 m (winter rapeseed 800,000 Plant/ha), and 1.0 m × 1.0 m (winter rapeseed 700,000 Plant/ha) intercropping, respectively. The lowercase letters in the columns represent the significant levels of the intercropping and control treatments at different stand densities (LSD, *p* < 0.05), and the uppercase letters represent the significant levels of the slope treatments (LSD, *p* < 0.05). ns indicates no significant difference, * indicates a significant difference at the *p* < 0.05 level.

## Data Availability

The original contributions presented in this study are included in the article. Further inquiries can be directed to the corresponding author.

## References

[1] Luo B., Geng P. (2024). New quality productivity of agriculture: Theoretical context, basic core and improvement path. Agric. Econ. Issues.

[2] Zou X., Zhu X., Chen C., Liu W. (2020). Soil and water conservation benefits of agroforestry system. Yunnan Univ. (Nat. Sci. Ed.).

[3] Pan K., Guo L., Chen X., Xu L., Tang J. (2022). Research progress on service functions of agroforestry ecosystem. J. Ecol. Rural Environ..

[4] Luo H., Wu T., Meng X., Liu P., Xu Y., Lin Z. (2024). Impact of forest-drug complex management model on soil ecology. World For. Res..

[5] Wang W., Huang G., Ren Y., Liu C., Jin Y., Liu C., Tang J. (2023). Plant growth and biomass dynamics of rubber-Rauwolfia-Cinnamomum cassia composite forest. J. Cent. South Univ. For. Technol..

[6] Surki A., Nazari M., Fallah S., Iranipour R. (2020). Improvement of the soil properties, nutrients, and carbon stocks in different cereal–legume agroforestry systems. Int. J. Environ. Sci. Technol..

[7] Huang J., Pan J., Zhou L., Yuan S., Lin W. (2020). Effects of light deficit on crop productivity in rubber-crop agroforestry system. Chin. J. Eco-Agric..

[8] Souza D.D.E.F., Oliveira S.D.N.D.J., Santos D.C.R.C., Ferreira O.D.V.E., Silva D.L.T.R., Paula D.T.M., Alves N.D.J., Oliveira D.R.S.J., Rodrigues M.D.I.J., Martins R.B.W. (2025). Physical and chemical soil quality and litter stock in agroforestry systems in the Eastern Amazonia. Agric. Ecosyst. Environ..

[9] Xie T., Shan L., Zhang P. (2022). Litter decomposition characteristics of poplar-maize composite system under different water conditions. Acta Ecol. Sin..

[10] Fitra Y.A.A., Oakley S., Prayogo C., Sari R.R., Saputra D.D., Ishaq R.M., Wicaksono S.K., Suprayogo D. (2024). Soil Water in Different Management Systems of Coffee-Pine Agroforestry and Its Relation to Coffee Bean Yields. IOP Conf. Ser. Earth Environ. Sci..

[11] Kun Á., Simon B., Zalai M., Kolozsvári I., Bozán C., Jancsó M., Körösparti J.T., Kovács G.P., Gyuricza C., Bakti B. (2023). Effect of Mulching on Soil Quality in an Agroforestry System Irrigated with Reused Water. Agronomy.

[12] Zhao L., Zhang Z., He H. (2025). Effects of phosphate fertilization and intercropping on plant growth, nitrogen and phosphorusuptake of millet and soybean across three soil types. J. Soils Sediments.

[13] Liu Y., Huang L., Liu Q., Li Z., Liu C., Yuan J., Liao J., Luo L., Yu C., Feng Y. (2024). Effects of tomato-Sedum alfredii Hance intercropping on crop production and Cd remediation as affected by soil types. Environ. Sci. Pollut. Res..

[14] Kokila A., Nagarajaiah C., Hanumanthappa C.D., Shivanna B., Sathish K., Mahadevamurthy M. (2024). Effect of Tree Canopy Cover on Soil Moisture Dynamics in Different Agroforestry Systems under Semi-arid Condition. Int. J. Environ. Clim. Change.

[15] Huo G. (2021). Interspecific Water Relationship and Influencing Mechanism of Apple-Rapeseed Complex System in the Loess Hilly Region.

[16] Narteh E.T., Adutwum A.A., Oppong H.T., Nketiah J.B., Pinamang P.A., Kwame A.T., Evans D., Vincent L., Olivia A., Ama S.E. (2019). Rubber and plantain intercropping: Effects of different planting densities on soil characteristics. PLoS ONE.

[17] Huang N., Li Y.X., Liu Y.H., Tang J.N., Ji Y., Zhao C., Li J. (2024). Effects of stand structure on ecological benefits and tree growth of Haloxylon ammodendron plantations: A meta-analysis. Land Degrad. Dev..

[18] Tkach V., Tarnopilska O., Luk’yanets V., Musienko S., Kobets O., Rumiantsev M., Bondarenko V. (2024). Density optimization of pine plantations in the Left-Bank Steppe in ukraine. Folia For. Pol..

[19] Lee J.-G., Lee D.-H., Jung J.-Y., Lee S.-G., Han S.H., Kim S., Kim H.-J. (2023). The Effects of Stand Density Control on Carbon Cycle in Chamaecyparis obtusa (Siebold and Zucc.) Endl. Forests. Forests.

[20] Biloa B.J., Monique A., Giweta H.M., Fiaboe M.K.K., Samuel N.N., Mandah V.P., Essobo D.J., Onana A., Cargele M. (2025). Influence of cocoa farm age and slope, and shade rate on cocoa soils fertility. Environ. Chall..

[21] Zhang L., Li P., Hu X., Zhong J., Yang K., Zhao Z., Li T. (2024). Response of loess with different slopes and vegetation coverage to rainfall infiltration. J. Soil Water Conserv..

[22] Xiao Z., Ai Q., Jin Z., Zhang B., Wang Y., Zhu Y. (2021). Study on the growth rhythm and dynamic changes of essential oil in Cinnamomum camphora dwarf forest. J. Jiangxi Agric. Univ..

[23] Jin Z., Zhang B., Ai Q., Xiao Z., Wang Y., Zhang H., Chen S., Lu Q., Wang Z. (2020). Necessity and feasibility analysis of research on camphor tree. J. Nanchang Inst. Technol..

[24] Lu J., Ren T., Li X., Cong R., Lu Z., Zhang Y., Liu S., Liao S., Zhu J. (2023). Precision regulation strategy for rapeseed nutrients =Strategy and efficient fertilization technology system. J. Huazhong Agric. Univ..

[25] Wang J., Zhang J., Yue J., Fu Z., Yin R., Chen Y. (2017). Effects of sodium chloride stress on photosynthetic pigments and chlorophyll fluorescence parameters of camphor tree seedlings. J. Anhui Agric. Univ..

[26] Luo X., Lu X., Zhang H., Han X., Xie R. (2024). Effect of planting density of camphor dwarf forest on rill morphological characteristics on red soil slope. Chin. J. Soil Water Conserv..

[27] Yao Y., Zhou J., An L., Kafle G., Chen S., Qiu L. (2018). Role of soil in improving process performance and methane yield of anaerobic digestion with corn straw as substrate. Energy.

[28] Yang C., Chen Y., Sun W., Zhang Q., Diao M., Sun J. (2024). Extreme soil salinity reduces N and P metabolism and related microbial network complexity and community immigration rate. Environ. Res..

[29] Nanjing Institute of Soil Science, Chinese Academy of Sciences (1978). Soil Physical and Chemical Analysis.

[30] Liu X., Yang Y., Wu B., Lv C., Wei H., Gao P., Zhang H., Dai Q., Chen Y. (2025). Effects of Nitrogen Application on Crop Production and Nitrogen Use in Rice–Wheat Rotation. Agronomy.

[31] Cheng B., Liu W., Wang L., Xu M., Qin S., Lu J., Gao Y., Li S., RAZA A., Zhang Y. (2021). Effects of planting density on soybean photosynthesis, yield and stem lodging resistance under corn-soybean strip intercropping. Chin. Agric. Sci..

[32] Wang K., Na E., Zhang R., Gao S., Liu J. (2021). Carbon, nitrogen and phosphorus stoichiometry and nutrient reabsorption characteristics of Pinus sylvestris var. mongolica in sandy land under different densities. J. Ecol..

[33] Li Q., Liu Z., Jinze G. (2022). Impacts of stand density on tree crown structure and biomass: A global meta-analysis. Agric. For. Meteorol..

[34] Li D., Yang Z., Yan P., Ling T., Qin F., Li P. (2024). Effects of stand density on canopy structure and understory herbs in Masson pine plantation community. J. Southwest For. Univ..

[35] Lott J.E., Ong C.K., Black C.R. (2009). Understorey microclimate and crop performance in a Grevillea robusta-based agroforestry system in semi-arid Kenya. Agri For. Meteorol..

[36] Zhang Y., Zhang N., Xu J., Xu D., Cheng F., Zhang C., Wu B., Gong Y., He Y., Wei S. (2024). Effects of different strip intercropping patterns on cotton growth, development and productivity. Chin. Agric. Sci..

[37] Xiang H., Zheng D., He N., Li W., Wang M., Wang S. (2021). Research progress on the physiological response of plants to low temperature stress and the effect of exogenous abscisic acid on stress alleviation. Acta Pratacult Sin..

[38] Wang H., Xiang Y., Li W., Shi H., Wang X., Zhao X. (2023). Estimation of aboveground biomass of winter rapeseed based on multispectral remote sensing of unmanned aerial vehicle. Trans. Chin. Soc. Agric. Mach..

[39] Li X., Wang Z., Bao X., Yang S., Wang P., Wang C., Wu J., Liu X., Tian X., Wang Y. (2021). Long-term increased grain yield and soil fertility from intercroping. Nat. Sustain..

[40] Han B., Xiao Z., Fu S., Wang L. (2014). Study on carbon storage of poplar-agricultural composite system with different densities. J. Anhui Agric. Univ..

[41] Cui J., Li S., Baoyin B., Guo D., Zhang L., Gu Y. (2024). Maize/Soybean Intercroping with Straw Return Increases Crop Yield by Influencing the Biological Characteristics of Soil. Microorganisms.

[42] Wang J., Bi H., Sun Y., Duan H., Peng R. (2018). Analysis of canopy shading range of single apple trees of different tree ages. Chin. J. Soil Water Conserv..

[43] Duan Z., Liu T., Zhang Y., Jiao C., Luan P., Yang T., Shi Y., Tian Y., Zhang W., Li L. (2017). Effects of distance between trees on photosynthetic characteristics and yield of wheat in intercropped jujube and wheat. J. Wheat Crops.

[44] Wang L., Dai Y., Zhang J., Meng P., Sun S., Li H., Wan X. (2020). Effects of water and light conditions on photosynthesis and growth of soybean in walnut-soybean agroforestry system. For. Sci..

[45] Lu X., Yang B., Zhang H., Zhang J., Wang Q., Jin Z. (2023). Inversion of essential oil yield of dwarf camphor tree leaves based on multispectral remote sensing of UAV. Trans. Chin. Soc. Agric. Mach..

[46] Zhao J., Fan H., Zhang J., Xiao Z., Zhang H., Xiao C., Wu C., Jin Z. (2020). Effects of slope position on biomass spatial allocation and essential oil yield of Cinnamomum camphora dwarf forest. J. Cent. South Univ. For. Technol..

[47] Zeng X., Li J., Xu M., Gao J., Sun N. (2006). Fertility status of red soil dryland and the influence of fertilization and utilization methods. Soil Bull..

[48] Liu Y., Liu Y., Lan Z., Tie N., Zhang M., Wang C., Luo Q., Zhang C. (2022). Effects of different irrigation methods on growth, photosynthetic characteristics and soil water transport of *Pinus sylvestris* var. *mongolica*. J. Nanjing For. Univ. (Nat. Sci. Ed.).

[49] Eduardo C., Míriam P., Jaime C., Javier G.D.D., Carla F., Lluís C. (2023). Close-to-nature management effects on tree growth and soil moisture in Mediterranean mixed forests. For. Ecol. Manag..

[50] Zhao Y., Qi X., Ren W., Che Z., Ren L. (2024). Research on technical measures for green manure crop rotation and fallow in rain-fed farmland in semi-arid areas. Arid Land Resour. Environ..

[51] Quan W., Jiu G., Meng Z., Zhang B., Rao Y., Xiao H. (2022). Nitrogen Reduction Combined with Organic Materials Can Stabilize Crop Yield and Soil Nutrients in Winter Rapeseed and Maize Rotation in Yellow Soil. Sustainability.

[52] Lu H., Xu J., Li G., Ma N., Su G., Zhang Y. (2024). Dynamic trends and laws of soil physicochemical properties and understory plant diversity in different growth and development stages of Eucalyptus grandis forest. J. For. Sci..

[53] Zhu Y., Zhai B., Sun M., Luo L., Wang Y., Du S. (2023). Soil physicochemical properties and stoichiometric characteristics of Robinia pseudoacacia and Pinus tabulaeformis plantations with different densities in the Loess Hilly Region. J. Soil Water Conserv..

[54] Lou Q., Chen H., Ding G., An N. (2022). Effects of afforestation density on understory vegetation and soil properties of Masson pine plantations. J. West For. Sci..

[55] Farooq T.H., Kumar U., Yan Y., Arif M.S., Shakoor A., Tayyab M., Rathod P.H., Altaf M.M., Wu P. (2022). Receptiveness of soil bacterial diversity in relation to soil nutrient transformation and canopy growth in Chinese fir monoculture influenced by varying stand density. Trees.

[56] Zhou H., Xiong J., Wu J., Zheng X., Huang H., Chen H., Yu X. (2023). Root spatial distribution and underground competition in a typical betel nut-elephant grass complex system in central Hainan. J. Trop. Crops.

[57] Ruan Y., Ou Z., Luo M., Yang T., Zhou Z., Hu G. (2024). Effects of rainfall level and planting pattern on slope runoff and sediment production in the metamorphic rock area of Dabie Mountains. Bull. Soil Water Conserv..

[58] Huo G., Zhao X., Gao X., Wang S., Pan Y. (2017). Soil water use and competition in the jujube-agriculture complex system in the Loess Hilly Region. J. Nat. Resour..

[59] Nguyen X.H., Pham A.H. (2018). Assessing soil erosion by agricultural and forestry production and proposing solutions to mitigate: A case study in Son La Province, Vietnam. Appl. Environ. Soil Sci..

[60] Zhang Y., Wang X., Wang Y., Yuan X., Li Y., Wang K. (2023). Ecological stoichiometric characteristics of Cynodon dactylon and its seedlings on different slopes in the Three Gorges drawdown zone. Acta Ecol. Sin..

[61] Wang B., Liu G. (1999). Effect of topography on soil nutrient loss in the loess hilly region. J. Soil Eros. Soil Water Conserv..

[62] Zhidan W., Xuan W., Tieliang W. (2022). Effects of Imazethapyr on Soybean Root Growth and Soil Microbial Communities in Sloped Fields. Sustainability.

[63] Nong G., Lu P., Wang L., Wu Q. (2013). Study on the distribution characteristics of wheat root system in sloping cultivated land on the Loess Plateau. Soil Water Conserv. Res..

[64] Yang Z., Liu Y., Chen M., Wang X., Ye C., Li X., Chen W., Yang Y., Wang B., Li C. (2022). Influence of Coupling Effects between Gravel Soil Porosity and Cement Grout Weight on Diffusion Laws and Morphologies of Penetration Grouting. Appl. Sci..

[65] Tu A., Nie X., Li Y., Li H. (2021). Experiment and simulation of water infiltration characteristics of layered red soil. J. Drain. Irrig. Mach. Eng..

[66] Lü W., Xiao F., Zhang S., Zheng W., Huang T., Xiao X., Li Y., Wu Y., Han D., Xiao G. (2020). Effects of seed and fertilizer application methods on rapeseed yield and fertilizer utilization efficiency in red dryland soil. Acta Agron. Sin..

[67] Zhang J., Cai D., Yang S., Chen X., Chen J. (2021). Effects of different slopes on soil fertility characteristics in Danjiangkou Reservoir area. Chin. Soil Fertil..

[68] Tang J., Yang Q., Liang J., Wang H., Yue X. (2024). Water management, planting slope indicators, and economic benefit analysis for Panax notoginseng production decision under shaded and rain-sheltercultivation: A three-year sloping fields experiment. Agri. Water Manag..

[69] Zhou Y., Dai C., Liu Y., Wang T., Deng J., Li Z., Hu Y. (2019). Effects of tillage measures and rainfall intensity on erosion of red soil sloping farmland in southern China. J. Soil Water Conserv..

[70] Chen X., Yang J., Tang C., Zheng T., Li L. (2017). Effects of rainfall intensity and slope on surface runoff and intersoil flow in red soil sloping farmland. Trans. Chin. Soc. Agric. Eng..

